# Impact of a Culturally Tailored mHealth Medication Regimen Self-Management Program upon Blood Pressure among Hypertensive Hispanic Adults

**DOI:** 10.3390/ijerph16071226

**Published:** 2019-04-06

**Authors:** Jessica Chandler, Luke Sox, Kinsey Kellam, Lauren Feder, Lynne Nemeth, Frank Treiber

**Affiliations:** College of Nursing, Medical University of South Carolina, Charleston, SC 29425, USA; soxl@musc.edu (L.S.); kellam@musc.edu (K.K.); feder@musc.edu (L.F.) nemethl@musc.edu (L.N.); treiberf@musc.edu (F.T.)

**Keywords:** mobile health, health disparities, medication adherence, hypertension

## Abstract

*Background:* Uncontrolled hypertension (HTN) and medication nonadherence are more prominent among Hispanics compared to non-Hispanic whites and African Americans. Advances in wireless health technology enable real-time monitoring of medication adherence (MA) and blood pressure (BP), facilitating timely patient–provider communication including tailored reinforcement/motivational feedback to patients and quicker titration changes by providers. The purpose of the current study was to conduct a 9-month smartphone-enabled efficacy trial addressing MA and BP control among Hispanic adults with uncontrolled HTN and poor MA. *Methods:* The research design was a 9-month, two-arm efficacy trial including an experimental (Smartphone Med Adherence Stops Hypertension, SMASH) group and an enhanced standard care (ESC) group. SMASH participants utilized a SMASH app which interfaced with a Bluetooth-enabled BP monitor for BP self-monitoring and an electronic medication tray. The ESC participants received text messages including links to PDFs and brief video clips containing healthy lifestyle tips for attention control. *Results*: Participants were 54 Hispanic adults (mean age: 46.5 years) with uncontrolled HTN. They were randomly assigned to either the SMASH (*n* = 26) or ESC group (*n* = 28). At baseline, no participants had controlled systolic BP (SBP). Baseline group averages for SBP between the SC and SMASH groups did not differ (150.7 and 152.3 mmHg, respectively; *p* = 0.53). At the 1, 3, 6, and 9-month time points, SBP averages were significantly lower in the SMASH versus SC groups (month 1: 125.3 vs. 140.6; month 3: 120.4 vs. 137.5, month 6: 121.2 vs. 145.7 mmHg; month 9: 121.8 vs. 145.7, respectively; all *p*-values <0.01). At months 3, 6, and 9 there was a significant difference between the percentage of participants meeting the 7th Joint National Committee cutoffs for SBP control in the SC and SMASH groups (month 3: 62.5 vs. 92.0%; month 6: 57.9 and 94.4%, month 9: 27.8 and 92.3%, respectively; all *p*-values ≤0.01). Average medical regimen adherence, as indicated by timestamped medication intake and BP monitoring for the SMASH group, ranged from 89.1 to 95.2% across the 9-month trial. *Conclusion*: Our findings indicate that our culturally tailored smartphone-enabled medical regimen self-management program may be an effective solution for the promotion of MA, resulting in statistically and clinically significant reductions in SBP among Hispanic adults with uncontrolled HTN.

## 1. Introduction

Essential hypertension (EH) affects one third of U.S adults and is a primary risk factor for stroke, cardiovascular events, and chronic kidney disease. [1] Hispanics, especially younger adults (<60 years old), have higher rates of uncontrolled EH compared to non-Hispanic whites and African-Americans (~62% vs. ~44% and ~59%, respectively) [2,3]. Medication nonadherence (MNA), the leading modifiable behavior responsible for uncontrolled blood pressure (BP), tends to be highest in persons with Hispanic ethnicity versus non-Hispanic whites and African-Americans [4] based upon self-report (e.g., 47% vs. 25.4% and 38.4%, respectively [5]) and pill counts (e.g., medication possession ratios: 0.61 vs. 0.82 and 0.68, respectively [6]). Hispanics’ alarming rates of uncontrolled EH, high MNA, low levels of consistent medical care, and high rates of EH-related cardiovascular and cerebrovascular disease as well as mortality highlight the need for user-centered, theory-guided, culturally attuned, medical regimen self-management programs which can foster BP control [6,7].

Three review articles totaling 133 EH randomized controlled trials (RCTs) involving uncontrolled hypertensives confirmed that strategies including BP self-monitoring, MA reminders (phone calls, SMS messages), and education programs/counseling resulted in small but statistically significant BP reductions. However, only 40–50% of participants achieved BP control, and BP improvements often deteriorated following cessation of the intervention [8,9,10].

Mobile health (mHealth), the application of wireless technology to health care, offers a highly scalable means of implementing these strategies. However, mixed findings (e.g., MA or BP control) were reported with some mHealth disease management programs. For some patients, providing a smart phone/tablet-enabled solution (e.g., applications interfaced with peripheral devices such as blue-toothed BP monitor, glucometer, electronic medication trays or vials, SMS-delivered MA/BP monitoring reminders, etc.) is adequate for initiating and sustaining desired behavior change (e.g., MA, BP self-monitoring) and clinical outcomes (e.g., improved BP control). However, others may require more motivational support to consistently use the mHealth self-management solutions, and/or greater integration of health care providers (HCPs) as part of the mHealth solution [11,12,13,14].

Among efforts directed at increasing patient motivation to sustain engagement in their medical regimen, incentive-based approaches (e.g., financial or token rewards, gamification) have had success in facilitating engagement in behavioral change programs (e.g., weight loss, MA to warfarin) [15,16,17].

A recent RCT with post myocardial infarction patients involved use of electronic pill bottles with daily lottery-based financial incentives for MA along with social support from family members/caretakers and feedback/social reinforcement from research staff to enhance MA and reduce subsequent cardiovascular events. Unfortunately, there were no significant differences between the mHealth group and standard care groups in MA nor subsequent hospitalizations. A three-month RCT with cardiometabolic patients found high and low daily lottery-based financial incentive conditions resulted in higher adherence than a control group in using a set of wireless biometric devices including a BP monitor. However, no significant differences were noted in changes in BP between the no-incentive control group and the two financial incentive groups.

Sustained engagement in various health behavior change programs (e.g., smoking cessation, diet, physical activity) including MA have been observed in interventions guided by several principles of Self Determination Theory (SDT) [18,19,20,21]. The first principle involves development of competency, akin to self-efficacy in which the individual develops the belief in their ability to engage in the desired behaviors. The second principal involves fostering increasing autonomous motivation to sustain these behaviors over time. SDT conceptualizes a continuum of human motivational regulation, ranging from fully external to internal [18,19,22]. External regulation, a form of controlled motivation, includes extrinsic rewards and punishment. Examples are financial incentives/constraints and pressure from others to change (e.g., family members, friends, healthcare providers). While external regulation may motivate short-term change, such changes are frequently less enduring and stable. The most powerful form of motivation is autonomous motivation. Here, one not only sees importance of one’s actions, but also links the new behaviors with their other core values, beliefs, and short and long-term life goals. Autonomous motivation in SDT relates to our need to feel independent in our actions rather than dealing controlled or coerced by others [23]. We attempted to promote increases in autonomous motivation by linking participants’ behavioral changes (i.e., increased MA) to their values, beliefs and goals, including specific motivational domains of family, faith, friends, community and work. Autonomously motivated individuals exert more effort and persistence in their behavior change, which is vital for managing chronic diseases due to lifelong nature of the medical regimen behaviors. Numerous studies have shown health behavior changes (e.g., physical activity, diet, smoking cessation, MA) have been more likely to be sustained across extended time periods (>12 months) in programs that focused upon fostering both self-efficacy in being able to engage in the protocols and increasing levels of autonomous motivation to sustain engagement in the desired behaviors [18,19,20,21,22,23].

We incorporated an iterative design process to develop, evaluate, and refine a culturally attuned, SDT-guided, patient and provider-centered mHealth medical regimen program for Hispanics [24,25,26]. The result was a smart phone application interfaced with several types of cellular-connected electronic medication trays and pill bottles, which provide reminder signals for MA, and SMS reminders to monitor BPs with a Bluetooth-enabled BP device. Timely tailored motivational and reinforcement SMS messages were sent based upon levels of MA. The MA feedback messages are tailored based upon responses to a values, beliefs, and goals (VBG) survey assessing one’s values, beliefs, and short/long-term life goals.

During the iterative design process, we developed and continuously refined our VBG branch logic survey by reviewing SDT-guided health behavior change programs, which identified participants’ values, beliefs, and goals associated with making health behavior changes (e.g., dietary changes, physical activity, smoking cessation, MA) [19,27,28,29]. We identified common motivational domains across the studies, which we have characterized as family, faith, friends, community and work. We also included assessment of short and long-term life goals, which are linked to one or more of the motivational domains.

The VBG survey includes five motivational domains: family, faith, friends, community activities/hobbies, and work. For each motivational domain, there are 5–8 branch logic questions. Within the family domain, respondents may indicate family history of chronic kidney disease, which would then lead to a more specific branch logic question regarding their own personal concerns of developing that disease in the future. Affirmative responses to the final branch logic question within specific domains creates the pool of possible motivational reinforcement text messages that may be sent for that participant based upon three grades of adherence (i.e., complete nonadherence, partial adherence, and complete adherence). For this project, each of the five motivational domains had a pool of 120 motivational messages broken into approximately 40 for each MA gradation. Each participant’s final pool, including number and content of messages, is dependent upon responses to this survey. For example, a single Hispanic man who was concerned that he would develop chronic kidney disease and have a stroke like his father and grandfather and was hoping to find a wife and have children could receive the following SMS for 100% MA: “You are doing great! Your family history doesn’t have to be in your future”. For partial MA: “Taking your meds is good. Taking them on time helps your heart, kidneys and brain even more!” For total MNA: “Get back on track taking your meds for building that stronger, healthier body for when you meet that special woman”. A three month proof-of-concept trial found the Smartphone MA Stops Hypertension (SMASH) program acceptable and feasible among Hispanic uncontrolled EH adults (100% retention rate, 97.5% MA, 89.2% BP monitoring adherence, mean SBP change of –47 vs. –12 mmHg for the standard care group) [24]. The purpose of the current study was to assess efficacy of the SMASH for Hispanics program in establishing SBP control via increased MA across a 9-month period among Hispanics with poor MA and uncontrolled EH.

## 2. Materials and Methods

### 2.1. Research Design

The SMASH study was a two-arm small-scale efficacy RCT. There was an experimental (SMASH) group and an enhanced standard care (ESC) group. The Institutional Research Board of the Medical University of South Carolina approved the study (Pro00034130). All participants provided written informed consent.

### 2.2. Participants

Inclusion criteria to participate were: Hispanic/Latino women or men, 21–65 years old, diagnosed with and prescribed medication(s) for EH, medication possession ratios <0.85, and systolic BP (SBP) >140 mmHg on last clinic visit within 12 months and at an initial BP screening evaluation. SBP was used as the selection variable since most EHs <65 have systolic or combination systolic/diastolic EH and for most patients, controlling SBP also results in diastolic blood pressure (DBP) control [30,31].

Exclusion criteria were inability to use a smartphone, a Bluetooth BP device, and electronic medication tray. Also, untreated psychiatric illness, alcoholism or substance abuse, medical diagnoses besides type 2 diabetes mellitus (e.g., cancer treatment within past two years, stroke, myocardial infarction), pregnancy, lactation or intention to become pregnant during the trial, participation in another clinical trial, and inability to speak, hear, or understand English or Spanish were criteria for exclusion. Spanish-speaking research staff contacted patients via telephone to determine interest and scheduled the initial BP screening visit with informed consent. Details of the BP protocol used at initial screening, all subsequent clinic evaluations, and by the SMASH mHealth group for their BP self-monitoring program are published elsewhere [24].

### 2.3. Protocol/Intervention/Groups

#### SMASH Intervention Group

SMASH involved a smart phone application compatible with Android or iOS platforms, the global systems for mobile (GSM) electronic medication tray Maya (MedMinder, Inc., Newton, MA, USA), and a Bluetooth-enabled BP device (AND model UA-767PlusBT, A&D, San Jose, CA, USA). The BP protocol for each SMASH participant was to measure their BPs every three days using the Bluetooth monitor paired with the SMASH app. SMS messages were sent to remind SMASHers to take their BPs every 3 days in the morning and evening. The SMASH app’s BP module provided options for use of audio instructions and/or text instructions with schematics illustrating correct placement of BP cuff, alerts when to take a BP reading (three readings with 5-min rest after first reading followed by a 2-min rest and then a final reading). A countdown timer was provided in between readings. Immediately after each reading, the user received audio and visual feedback of their systolic and diastolic BP and heart rate levels. The app also provided a cumulative table of average BPs displayed in categories of daily, weekly, and/or monthly BP progress reports. The BP data were securely transferred to our Health Insurance Portability and Accountability (HIPAA)-compliant servers for processing with timestamps providing information for calculation of adherence levels to the BP protocol. This adherence score is what drove the BP-related reminder and feedback messages. SMS messages were also used to deliver automated tailored motivational/social reinforcement messages based upon MA levels. These messages were based upon the participant’s responses to a branch logic questionnaire, which identified their values, beliefs and short/long term life goals. During the iterative design process [23,24,25], with input from Hispanic clinical research staff and Hispanic adults with hypertension, we developed a pool of over 600 messages, with ~200 messages for each MA gradation (non-adherence, partial adherence, and complete adherence). The tailored feedback messages were automatically chosen from the pre-populated pool of messages based upon: (1) each participant’s previous day’s adherence score, and (2) their responses to the branch logic questionnaire.

Medication trays provided a series of reminder signals to take medications (blinking light, intermittent chime, automated SMS/phone call). Initially, the frequency of SMS messages received was based on the previous day’s MA score. After 4 weeks of 100% MA, message delivery changed to a 3-day interval schedule. While we did not maintain a file of the total number nor the exact message content sent to each participant across the trial, we do not expect that our system duplicated messages, meaning we do not think a participant received the same message twice. Given the majority of our SMASH intervention arm reached 100% MA adherence within the first 2 months, our participants did not receive >200 messages (i.e., the number of unique messages per MA gradation) given that the message delivery schedule changed from daily to a 3-day interval after one month of 100% MA. These messages were intended to foster association of one’s increasing MA being linked to their core values, beliefs, and life goals, ultimately fostering greater sustained engagement via increased autonomous motivation to adhere to the regimen. The ESC group received comparable frequency of SMS messages which provided healthy lifestyle tips unrelated to MA (e.g., nonsmoking, increased physical activity, lower fat, sugar and sodium intake). These messages often had links to brief PDFs and short videos.

Importantly, the SMASH program was developed with significant involvement from healthcare providers. They set BP threshold ranges (BP > 180/105 or < 100/75 mmHg) for the home BP self-monitoring. If the cutoffs were exceeded, a designated HCP would be contacted in order to determine the course of action to take with the participant. They also crafted brief summary reports to be sent out every two weeks designating participants’ MA per week, total number of sessions per week, average SBP/DBP, and upper and lower BP levels for each week. Collectively, this information was used to make titration changes for the participants and in some cases to schedule office visits.

### 2.4. Outcome Measures

The primary outcome was change in resting systolic blood pressure (SBP) from baseline to the 6-month time point. Secondary outcomes were resting DBP and MA (using medication tray timestamped data).

## 3. Results

We recruited 56 Hispanic adults with a mean age of 46.5 ± 9.9; 61% men. Following randomization into the trial, two SMASH subjects were subsequently dropped due to unidentified exclusion criteria (alcoholism and congestive heart failure) resulting in a total sample of 54 participants (SMASH = 26 and ESC = 28). No statistically significant differences were observed between the SMASH and ESC groups across any of the demographic or baseline descriptive variables presented in Table 1 (all *p*-values > 0.80).

We observed an overall recruitment rate of 87% (54/62) among those who initially met all inclusion criteria. Of the eight who withdrew from the study, four cited not wanting to disclose personal background information. During the trial, three participants were withdrawn due to identification of previously unidentified exclusion criteria, alcoholism, congestive heart failure, and chronic kidney disease. One was lost to follow-up due to leaving the state.

### 3.1. Resting Systolic Blood Pressure Level Changes

**Figure 1:**
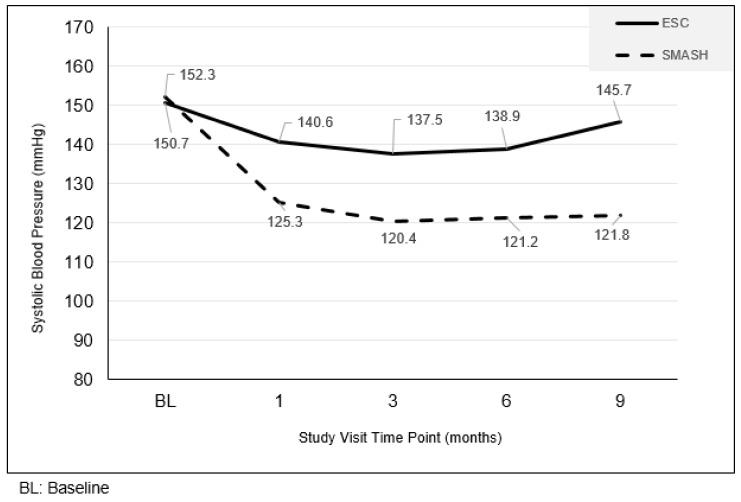
Changes in resting systolic blood pressure by study arm.

The primary outcome variable was changes in clinic based resting SBP. There were no statistically significant differences between the SMASH and ESC groups at baseline (*p* = 0.604). The SMASH group’s SBP was significantly lower than ESC group at each subsequent evaluation (Figure 1; all *p*-values <0.001). General linear mixed model (GLMM) analyses of treatment over time (baseline, 1, 3, 6, and 9 months) revealed a significant treatment by time interaction for SBP (*p* <0.001). Post hoc analyses indicated that at the 1, 3, 6, and 9-month time points, SBP averages were significantly lower in the SMASH versus SC groups (month 1: 125.3 vs. 140.6; month 3: 120.4 vs. 137.5, month 6: 121.2 vs. 138.9 mmHg, month 9: 121.8 vs. 145.7, respectively; all *p*-values < 0.01).

### 3.2. Resting Diastolic Blood Pressure Level Changes

**Figure 2:**
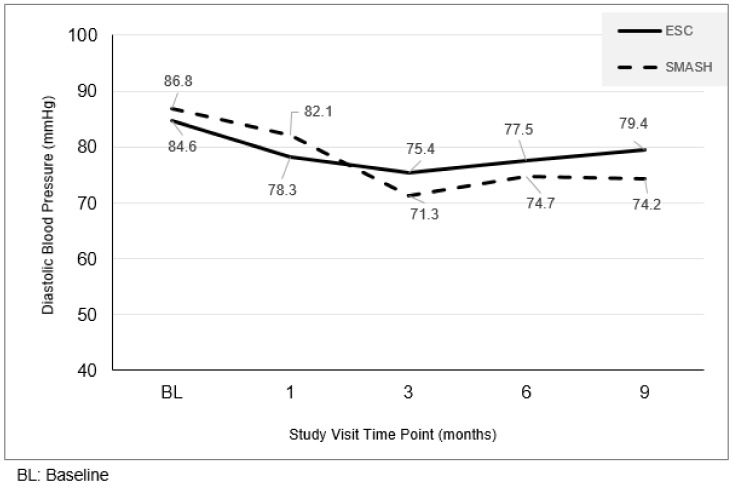
Changes in resting diastolic blood pressure by study arm.

Similar to the resting SBP findings, the SMASH and ESC groups were not significantly different at baseline (*p* > 0.49; see Figure 2). The GLMM analyses revealed a significant treatment by time interaction for DBP (*p* < 0.001). Post hoc analyses indicated that at the 1, 6, and 9-month time points, DBP averages were significantly lower in the SMASH versus SC groups (all *p*-values < 0.01).

### 3.3. The Seventh Joint National Committee (JNC7) Blood Pressure Control Changes

As expected and illustrated in Table 2, no subject met JNC7 SBP control (<140 mmHg) at baseline. Using Fischer exact tests, the SMASH group showed significantly higher percentages of SBP control at each subsequent time-point (all *p*-values < 0.01). At all-time points, there was a significant difference between the percentages of participants meeting JNC7 cutoffs for SBP control for the ESC and SMASH groups (month 1: 42.3 vs. 80%; month 3: 62.5 vs. 84.0%; month 6: 26.3 vs. 72.2%, and month 9: 27.8 vs. 92.3%, respectively; all *p* < 0.01). Approximately 60% of all subjects met DBP control (<90 mmHg) at baseline with SMASH group exhibiting higher, however not statistically significant, percentage DBP control at each evaluation (Table 3).

### 3.4. Medication and BP Self-Measurement Adherence

Timestamps of openings of pill tray compartments were used to calculate MA scores for the SMASH intervention arm participants using an algorithm fully described elsewhere [24]. Briefly, doses taken within a 3-h window of the predesignated time received a full score for that dose, those within a 3–6 h window received a score of 0.5 for that dose, and those taken after more than 6 h received a score of zero. The SMASH group’s average MA per month ranged from 0.89 to 0.95 (average = 91.0%). Importantly, after the 6-month evaluation if MA was 1.0 for that month and SBP was controlled, participants could opt to return the tray. Seventy-three percent of the eligible SMASH participants did so. All that returned the tray maintained good MA measured via self-report using the Morisky Medication Adherence Scale [32] (Table 4) and subsequent SBP control following tray return. The Morisky MA self-report findings indicated no significant differences between the SMASH and ESC groups at baseline. Subsequently, the SMASH group reported significantly greater increases in MA at each evaluation.

The SMASH participants also showed good protocol adherence to the BP self- monitoring using the provided SMASH app and Bluetooth BP device. The overall adherence for total expected readings was 1.00 ± 0.24. For adherence to the more stringent definition of “on-time BP adherence” rate for taking their BPs every 3 days, the SMASH group did so 85.7% of the time. They averaged 8.8 ± 5.7 intervals >3 days during the 184.1 ± 2 days they participated.

### 3.5. SMASH Satisfaction and Usability

The high levels of adherence to the medication schedule and BP self-assessment schedule indicated high levels of usability of the SMASH system including the smart phone app, Bluetooth BP monitor and the electronic medication tray. A random sample of 10 participants in the SMASH group were administered the telehealth satisfaction and usability questionnaire [33]. This 20-item questionnaire uses a 5 point Likert scale (1 = strongly disagree–5 = strongly agree). The average score total score (possible range = 20–100) was 98. Overall satisfaction with the SMASH system was an average of 4.87 and ease of use of the electronic tray and the smart phone app/BP monitor were both scored by all as a five.

## 4. Discussion

This two-arm efficacy RCT showed a user centered, culturally tailored, mHealth medication and BP self-management system (SMASH) quickly established and sustained BP control across a 9-month period among Hispanics with history of poor MA and uncontrolled hypertension. The SMASH intervention group compared to the enhanced standard care (ESC) group experienced earlier and much higher degrees of sustained SBP control based upon JNC7 guidelines (SBP < 140 mmHg). Both groups by definition based upon inclusion criteria were at zero levels of SBP control at baseline. The SMASH group climbed to 80% vs. 42.3% for the ESC group by month 1. They continued to increase SBP control levels and maintained >92% control throughout the remainder of the study. The ESC group reached a peak of SBP control at 3 months, and then continued to decline to a low of 27.8% at month 9. Interestingly, both groups exhibited significant improvements in DBP control (<90 mmHg). Both groups started out with ~60% meeting DBP control cutoff. The SMASH group maintained 100% DBP control throughout the trial while the values for the ESC group ranged from 88.5% to 94.7%. Collectively, these findings across the 9 months corroborate and extend not only our earlier work with Hispanics [24], but results of the SMASH program adapted for different ethnic groups and patient populations. Three other proof-of-concept and feasibility trials involving African Americans with sole diagnosis of uncontrolled hypertension, as well as African-Americans recovering from a recent stroke and African-American and non-Hispanic white kidney transplant recipients with uncontrolled hypertension showed comparable MA levels and BP control levels [24,25,34,35,36].

Compared to Xiong et al.’s 2018 review including eight MA studies directed at improving BP control in hypertensives [37], our program resulted in greater average reductions in SBP (30.5 vs. 9.6 mmHg), thus also achieving greater proportion of participants with SBP control. Interestingly, we did observe several similarities between our program and another highly successful program in reducing SBP [38]. Both studies included significant involvement of a HCP who received synthesized MA and biometric data reports. They were able to make clinical decisions much quicker than waiting for the next scheduled clinic visit more than three months away. This caveat may partially account for the inconsistency in mHealth program outcomes due in part to not monitoring both MA as well as associated biometric functioning, or if they do, not integrating the information into healthcare management process in a timely manner.

To date, the chronic disease management RCTs that have been conducted using various forms of digital medicine technology have had mixed results. Some studies have shown improvements in the primary clinical outcomes (e.g., JNC-defined BP control, Hemoglobin A1c < 7, MA > 0.85), but many others have not shown a significantly greater degree of improvement compared to standard care groups. [12,39,40,41] We noted earlier that critical to the success of medical regimen self-management programs is inclusion of objective measures of MA, relevant biometric markers associated with medications being monitored, and integrated involvement of HCPs in making clinical decisions based upon the data. Also critically important is that the patient correctly engages in various aspects of the regimen on a sustained basis. Individuals must have functional health literacy established related to the regimen behaviors and be motivated to engage. A critical ingredient in the success of the SMASH program involved using a patient and provider centered, iterative design process guided by the principles of Self Determination Theory [21,22]. As part of the iterative design process, we spent significant amount of time via focus groups and key informant interviews identifying motivational drivers for patients’ engagement in their medical regimens. Common domains across all ethnic groups and types of chronic diseases included individuals’ short- and long-term life goals (e.g., wanting to get married, have children, participate in grandchildren’s development, stay employed to reach goals such as purchase of a home, assist in college education cost of family members, have a stable retirement, etc.). We also identified motivational domains related to engaging in activities related to “*familisimo*”, faith (e.g., attending church, prayer meetings, bible studies, etc.), friends, recreational and community activities, and work [24]. Through post-trial interviews of the previous Hispanic proof-of-concept study [20], we found they voiced a need for providing timely tailored motivational and social reinforcement feedback related to their interest in these various domains linked to their degree of engagement in the regimen. That resulted in the generation of ~600 different SMS messages divided into three categories (complete, moderate, and nonadherence). By the 6-month mark, 73% of those whose blood pressure was controlled and who had 100% MA over the previous month elected to return their medication tray but continue monitoring their BP and using the SMASH app. Perhaps through the tailored SMS messages, they increased their competence and levels of autonomous motivation to continue their MA, as they all maintained JNC7 BP control and high medication possession ratios (MPRs) and Morisky scores.

Although the results are promising, this study is not without limitations. First, the ESC group did not use an electronic medication tray. This prevented direct comparison of date and time stamped derived MA scores between the groups. We did collect medication possession ratio data but found, as experienced in earlier trials, difficulties with some participants not keeping a record of what day and how many pills they added to a particular bottle from other available prescriptions of the same medication. With those in the SMASH arm that did not engage in such activity, we did find commensurate increases in MPR levels, as their Medminder tray-derived MA scores increased. The Morisky MA self-report questionnaire findings lent support that the ESC group did not show significant improvements in their MA, whereas the SMASH group did and mimicked the medication tray MA derived findings. Second, the ESC group showed improvements in SBP and DBP levels not nearly of the same magnitude as the SMASH group but which rival some digital technology-enabled intervention arm studies’ outcomes [37]. These improvements may possibly be due to several factors including clinical management contamination. The HCP network involved in this study provided all participants with prescriptions for most often free medications available from local grocery store pharmacy. Furthermore, over the course of the trial, several groups of participants were recruited who lived in the same rural farming camp. They often travelled together in a shared farm van to pick up their medications while getting groceries at a grocery store. This likely contributed strongly to the filling of the medication scripts. Lastly, follow-up appointments were scheduled well in advance. The ESC participants seemed to prepare accordingly based on their self-report during the clinical evaluation of having taken their medications a day or so in advance and on the day of the visit. This was confirmed through a question added our data collection document that asked, “What was the date and time of your most recent dose?” Additionally, the project manager anecdotally confirmed this through conversations with the participants who stated that they knew she was coming and they knew they should take their medications before she arrived for their scheduled appointments. The MPR and Morisky data provided a better overview of their day-to-day MA behaviors. Through MPRs we were able to verify that they did not take their medications every day. Morisky MA self-report questionnaire indicated slight increase in MA but nowhere near levels consistently reported by the SMASH group which were verified via their time stamped medication intake and reductions in SBP. However, without objective MA data, we are unable to confidently infer the cause of these BP improvements. Future studies should include the resources to collect objective MA data from all participants, including the control groups.

## 5. Conclusions

In conclusion, our findings indicate that our SMASH mHealth program, developed through a theory-guided, iterative, and patient-centered process, assisted patients in establishing sustained MA and BP self-monitoring behaviors. This led to statistically significant and clinically relevant BP reductions among Hispanic adults with a history of uncontrolled hypertension and poor MA. The combination of tactics which contributed to the success of this program included real-time measure of MA, relevant biometric self-monitoring, integrated HCP engagement to provide timely titration changes, fostering of functional health literacy via SMS messages and audio and video clips, and linkage of MA to patient-identified goals. With the continued transformation of our nation’s health care reimbursement system to one of pay-for-performance (e.g., preventing hospitalizations within 30 days of discharge, meeting best practice guidelines for risk factor levels, etc.), the Centers for Medicare and Medicaid Services (CMS) has increased the number of Current Procedural Terminology (i.e., CPT) codes to enable payment of remote healthcare monitoring within delivery of care. In order for the SMASH program to become competitive for selection by hospital administrators, practice plans, and insurance carriers, the SMASH program would benefit from application in a large-scale multi-site effectiveness RCT with various ethnic groups and patient populations.

## Figures and Tables

**Table 1:** Descriptive Characteristics of participants (*n* = 54).

Demographic		SMASH (*n* = 26)	ESC (*n* = 28)
Age (M+/- SD)		44.4 ± 7.2	46.8 ± 8.1
Gender	Male	29%	38%
	Female	71%	62%
Marital Status	Single	24%	10%
	Married/Living with Significant Other	47%	62%
	Separated/Divorced	24%	21%
	Widowed	5%	7%
Education	High School or less	71%	72%
	Partial/College Grad	29%	28%
Income	$0–25,000	71%	62%
	$25–50,000	29%	21%
	>$50,000	0%	3%
	Not Reported	0%	14%
Employment	Full/Part-Time	41%	69%
	Retired/Disabled	0%	0%
	Unemployed	59%	31%

M: mean; SD: Standard Deviation.

**Table 2:** Proportion of participants meetings the 7th Joint National Committee guidelines for-controlled systolic blood pressure (SBP < 140 mmHg).

Controlled SBP	SMASH	ESC	*p*-Value
Baseline	0	0	--
Month 1	80.0	42.3	0.005
Month 3	92.0	62.5	0.013
Month 6	94.4	57.9	0.009
Month 9	92.3	27.8	0.001

**Table 3:** Proportion of participants meeting 7th Joint National Committee guidelines for controlled diastolic blood pressure (<90 mmHg).

Controlled DBP	SMASH	SC	*p*-Value
Baseline	61.5	64.2	0.838
Month 1	100	88.5	0.083
Month 3	100	91.7	0.145
Month 6	100	94.7	0.337

**Table 4:** Morisky Medication Adherence Self-Report.

Enhanced Standard Care			SMASH			
Study Visit	Mean	SD	Mean	SD	∆	*p*-Value
Baseline	6.99	2.39	6.83	1.99	0.154	0.78
1	7.85	1.66	9.42	0.95	1.573	**<0.001**
3	7.98	1.95	9.13	1.50	1.142	**0.017**
6	7.48	1.7	10.22	0.43	2.741	**<0.001**
9	6.84	1.52	9.81	1.31	2.971	**<0.001**

**Bold text** indicates significant at the *p* < 0.05 level.
